# Malignant fungating wounds assessment in palliative care: a scoping review

**DOI:** 10.3389/fpubh.2025.1602493

**Published:** 2025-06-12

**Authors:** Daniela Nigrelli, Francesca Gambalunga, Giuliano Anastasi, Angela Peghetti, Stefano Durante, Martina Giusti, Valentina Biagioli, Silvio Quirini, Laura Iacorossi, Roberto Latina

**Affiliations:** ^1^Department of Biomedicine and Prevention, University of Rome Tor Vergata, Rome, Italy; ^2^Professional Health Care Services Department, University Hospital Policlinico Umberto I, Rome, Italy; ^3^Department of Trauma, AOU G. Martino University Hospital, Messina, Italy; ^4^SPIR - Professional Development and Research Implementation, IRCCS Azienda Ospedaliero-Universitaria di Bologna, Bologna, Italy; ^5^Strategic Steering Commitee, Centro Studi SAPIS Foundation, Italian National Federation of Orders of Radiographers and Technical, Rehabilitation, and Prevention Health Professions Research Centre, Rome, Italy; ^6^Department of Experimental and Clinical Medicine, University of Florence, Florence, Italy; ^7^Department of Medical and Surgical Sciences-DIMEC, University of Bologna, Bologna, Italy; ^8^Department of Life, Health and Health Professions Sciences, Link Campus University, Rome, Italy; ^9^Department of Health Promotion, Mother and Childcare, Internal Medicine and Medical Specialties, University of Palermo, Palermo, Italy

**Keywords:** malignant fungating wound, symptom assessment, wound healing, tool, neoplasms, scoping review, nursing

## Abstract

**Introduction:**

Malignant fungating wounds (MFWs) are secondary chronic wounds resulting from malignant cell proliferation and migration, compromising skin integrity in patients with cancer. These wounds present a range of signs and symptoms. Although several instruments are used in their assessment, it is still unclear which tool is most appropriate for comprehensive evaluation and wound healing.

**Aim:**

To review the existing instruments for MFW assessment, highlighting their strengths and limitations.

**Methods:**

A scoping review was conducted following the Arksey and O’Malley framework (2005), the Joanna Briggs Institute guidelines (2020, 2021), and the PRISMA-ScR checklist (2018). The search was performed on four databases: Web of Science, Scopus, PubMed, and EBSCO.

**Results:**

Forty studies were included, describing 22 instruments. They described half targeted general symptoms, and half wound-related signs and symptoms. Four instruments were specifically designed for MFWs, all based on the Malignant Wound Assessment Tool (MWAT). These were: MWAT – Clinical; MWAT– wound bed status; MWAT– Perception; MWAT – Research. However, only the Clinical and Research versions were validated in English, but neither was subjected to psychometric validation, and lacked a comprehensive assessment, such as key symptoms.

**Conclusion:**

Despite the existence of specific tools for MFW assessment, a comprehensive, validated, and standardized tool is still lacking. While the Clinical and the Research versions of the MWAT offer a broad assessment of MFWs, they require refinement to address overlooked symptoms and validation in other languages. Establishing standardized, multidimensional measures could enhance clinical decision-making and improve outcomes for patients living with MFWs.

## Introduction

Malignant fungating wounds (MFWs) are complex, secondary chronic wounds arising from the uncontrolled proliferation and infiltration of malignant cells, compromising skin integrity in patients with cancer (1). They can present as an ulcerated wound, raised nodules with a cauliflower appearance, or a combination of these forms, explaining the term ‘fungating’ used to describe these ulcers (2). MFWs can develop due to primary skin malignancies, like melanoma, basal cell carcinoma, or squamous cell carcinoma, direct spread from an underlying cancer, or when the tumor metastasizes and breaks the skin (3).

The epidemiology of MFWs remains uncertain due to limited data (4), but estimates suggest that 5–10% of cancer patients may develop such wounds, typically in the last 6 months of life, with minimal prospects of wound healing (1). MFWs are most frequently located on the breast (62–66%), head and neck (22–24%), or chest (1%) (5). These wounds are characterized by rapid tumoral growth, excessive exudate, necrosis, pruritus, malodor, and bleeding (6). Excessive exudate can result from increased vascular permeability, infection, or devitalized tissue (7), while necrotic tissue fosters bacterial proliferation, leading to secondary infections and malodor (8). Such odor can induce nausea, reduce appetite, drive social withdrawal, and contribute to depression among both patients and caregivers (9). Patients with MFWs experience a significant symptom burden, and studies have shown that managing these symptoms not only improves patient outcomes and physical well-being but also enhances their self-esteem (10). Bleeding commonly occurs due to friable tissue and impaired homeostasis, compounded by cancer disease and treatment that influence coagulation (7). Rapid tumor growth can also compress nearby structures, such as nerves and lymphatic vessels, leading to pain, reduced mobility, and impaired drainage. These issues can be worsened by incorrect wound dressing techniques (1, 2).

The numerous and distressing symptoms associated with MFWs can significantly affect the quality of life of patients with advanced cancer (11). Low-performance status and the challenges of ongoing wound care may further compromise essential dimensions of health, such as functional status, social relationships, and mental well-being (12, 13). Moreover, the terminal prognosis of MFWs imposes a substantial emotional burden on patients and their families, which in turn often results in patients experiencing fear and uncertainty about the future (14, 15).

Given these challenges, developing pragmatic, patient, and family-centered palliative wound care strategies is essential, beginning with a comprehensive assessment of MFWs (12). Such assessment can enable appropriate symptom evaluation, prioritizing patient safety, comfort, and quality of life (16). For instance, managing exudate and bacterial colonization through proper wound cleaning can be transformative for patients, alleviating discomfort, pain, and social isolation triggered by leakage of malodor (17). Nurses play a central role in the assessment of wounds, leveraging their expertise to identify complications early, implement personalized pain management strategies, and provide education and support to patients and their families (18). Their ability to assess wounds within the broader context of advanced illness enables them to make meaningful contributions to patient comfort and dignity (19). Therefore, an adequate assessment could support the palliative care nurses in managing these wounds, which is currently challenging given the heightened risk of complications (20). Although the literature highlights the clinical relevance of these symptoms, there are still gaps in accurately documenting their prevalence, characteristics, and impacts on patients’ functional status, underscoring the need for comprehensive assessment (21). However, to the best of our knowledge, no reviews have systematically evaluated the tools available for assessing and managing MFWs.

To address this gap, a clinical tool that facilitates the evaluation of MFW signs and symptoms is urgently required (7), both to guide interventions (22) and to overcome the barriers to evidence-based care (23). Such interventions should reflect patient and family goals of care, especially in advanced disease where the symptom burden can be invalidating, with physical disfigurement and emotional debilitation (24). Given the complexity of MFWs, it is also relevant to inform both patients and families about the scenarios, reassure them, and ensure that the healthcare providers can assess, select, and deliver tailored and appropriate treatment to best manage the condition (25). In light of these considerations, the present study seeks to answer the following question: What instruments are currently available for the comprehensive assessment of MFWs?

## Aims

The primary aim of this study is to review the existing research on the comprehensive instruments used to assess and manage malignant fungating wounds (MFWs). Specifically, we aim to (1) identify how current research addresses the assessment of MFWs’ signs and symptoms, and (2) highlight the strengths and limitations of available tools to guide clinical practice and future research.

## Materials and methods

This scoping review followed the five-stage framework of Arksey and O’Malley (26) and the methodology outlined by the Joanna Briggs Institute (JBI) (27, 28). We also adhered to the PRISMA-ScR (Preferred Reporting Items for Systematic Reviews and Meta-Analyses extension for Scoping Reviews) checklist to ensure transparent reporting (29).

### Eligibility criteria

In line with established scoping review methodologies (27–29), we formulated eligibility criteria and their justification. We included peer-reviewed quantitative and qualitative studies reporting on tools for MFW assessment, without limiting to specific symptoms or signs, to capture a broad range of characteristics. No temporal and language restrictions were applied, ensuring a comprehensive overview of the literature. Studies were excluded if they focused on other types of wounds (e.g., surgical), involved non-cancer populations, or lacked information on instruments relevant to MFW assessment. Gray literature and non-empirical research (e.g., editorial) were excluded to prioritize feasibility and ensure the inclusion of peer-reviewed tools, consistent with previous review (30). Eligibility criteria were refined iteratively, as recommended by Arksey and O’Malley (26), allowing for adjustment based on emerging insights from the literature.

### Information sources

An extensive search was performed in four databases to identify potentially relevant studies.

Pub-Med, Web of Science, Scopus, and EBSCO were used. These databases were selected for their broad coverage of biomedical, nursing, and health sciences literature, ensuring a comprehensive and multidisciplinary retrieval of studies related to MFWs’ assessment. The initial search was conducted from 06th July 2024 to 11th February 2025.

### Search strategy

Search strings were developed using a combination of controlled vocabulary (e.g., MeSH terms), free text keywords, and Boolean operators. Each search string was tailored to the specific databases consulted. Consistent with methodology, reference lists and citations of all retrieved full-text articles were screened to identify additional eligible studies. Zotero X8 software (Clarivate Analytics, Philadelphia, PA) was employed to organize records and ensure traceability. Duplicates were removed through the automated merging function of Zotero and manual checking. The final search strings used can be found in Table 1.

**Table 1:** Queries with keywords, Boolean operators, and Mesh terms were utilized for each database.

	Query	Database
#1	(fungating AND (wound* OR lesion* OR tumor* OR tumor* OR cancer* OR neoplas*)) OR (“ulcerating cancer*”)	PubMed
#2	(fungating OR (wound* OR lesion* OR tumor* OR tumor* OR cancer* OR neoplas*)) OR (“ulcerating cancer*”)	Web of Science
#3	(fungating OR (wound* OR lesion* OR tumor* OR tumor* OR cancer* OR neoplas*)) AND (“ulcerating cancer*”)	EBSCO and Scopus

### Selection of sources of evidence

The study selection followed a two-stage process. In the first stage, two authors (DN, FG) independently screened the titles and abstracts of all identified records against the inclusion and exclusion criteria. Records deemed potentially eligible were advanced to the second stage, and the full texts were retrieved. In the second stage, two other authors (GA, RL) reviewed the full texts to confirm their eligibility. Any disagreements in study selection were resolved through discussion with a third reviewer (AP), ensuring consensus on final inclusions.

### Data charting process

A data-charting form was created in Microsoft Excel following the JBI scoping review guidelines (27) to collect relevant data from the included studies. Two authors (DN, RL) independently extracted the data, and any discrepancies were resolved by consultation with another two authors (AP, SD) until an agreement was reached.

### Data items

The following information was extracted from each included study: reference (author and year of publication), country, study objective, study design, instrument used for MFWs assessment, symptoms considered, the database from which the study was identified, and the language of publication.

### Critical appraisal of individual sources of evidence

Considering that scoping reviews are generally undertaken to map existing evidence rather than evaluate methodological quality or risk of bias (29), we did not formally assess the quality of the included studies. Consequently, no study was excluded based on methodological limitations, ensuring a broad representation of the available literature. Nonetheless, every study included in this review was published in a peer-reviewed scientific journal.

### Synthesis of results

A narrative approach was used to synthesize and present the findings of this scoping review, in line with the framework by Arksey and O’Malley (26). Studies were organized based on whether they employed instruments explicitly designed for wound assessment or instead targeted general symptoms without a wound-specific focus. We also searched the databases consulted for this review for validation studies related to each identified instrument. The key findings are described narratively in the results section and supplemented with tables, graphs, and visual representations when appropriate.

## Results

### Selection of sources of evidence

**Figure 1:**
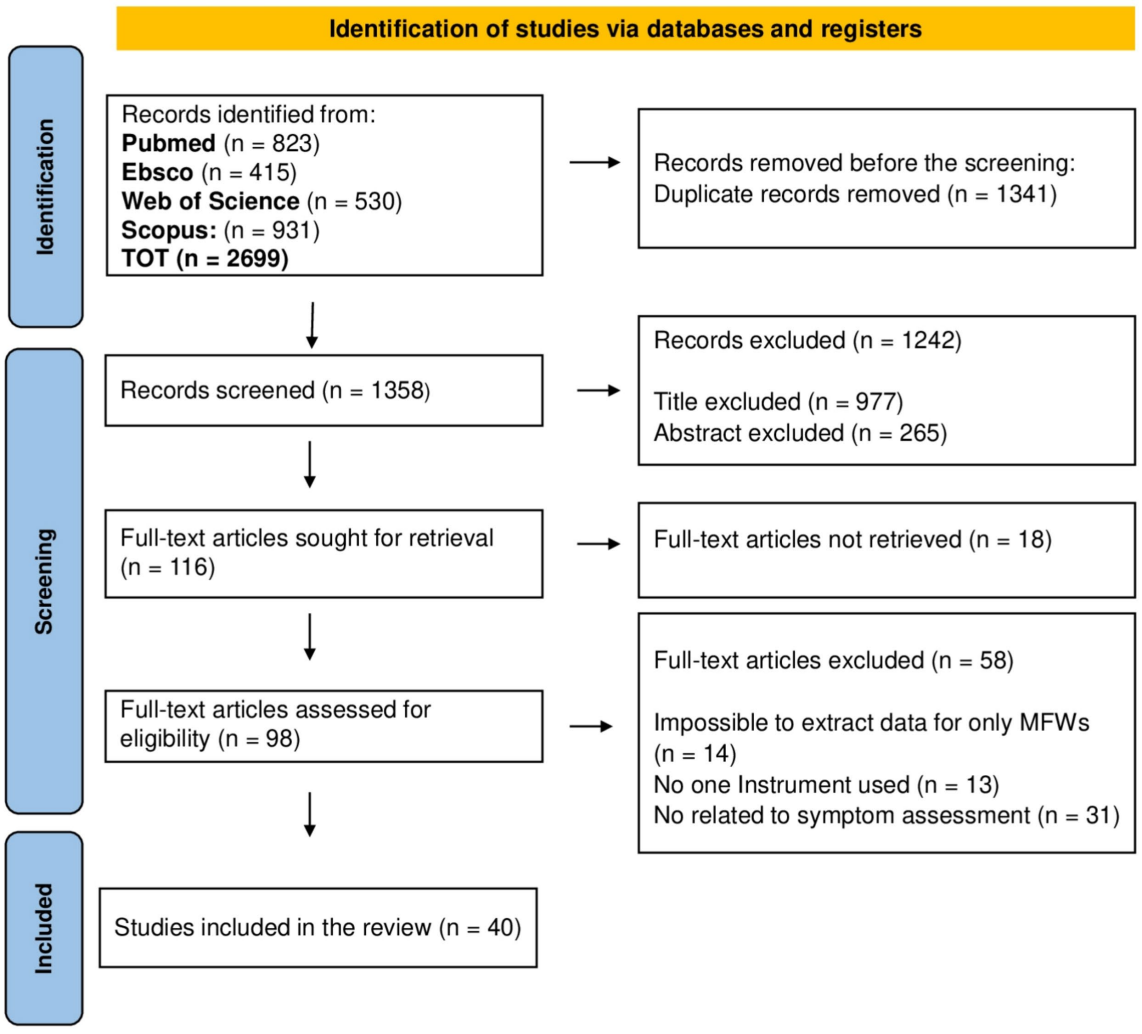
PRISMA flowchart illustrating the selection process and the number of sources included and excluded.

The search on the four databases returned 2,699 potentially relevant records. After the duplicates were removed, 1,358 records proceeded to the first stage of screening. After screening for relevance based on title and abstract, 116 records proceeded to the full-text screening phase. All identified full-text records were retrieved. Only 40 articles met the eligibility criteria and were included in the review. A PRISMA flow diagram (31) describing the screening process is displayed in Figure 1, with the number of included and excluded records for each phase and the justification for exclusion.

### Characteristics of sources of evidence

The characteristics of the studies included in this review are presented in Tables 2, 3.

**Table 2:** Characteristics of sources of evidence.

Reference	Country	Study objective	Study design	Database
Adderley and Holt (2014) (32)	United Kingdom	To review the literature on the effects of dressings and topical agents on MFW symptoms.	Systematic review	PubMed, Scopus, Web of Science
Agra et al. (2015) (33)	Brazil	To describe the palliative nursing care directed to a patient with basal cell carcinoma and MFW.	Case study	EBSCO
Agra et al. (2019) (34)	Brazil	To evaluate the knowledge and practice of nurses in managing pain in patients with MFWs.	Observational study	EBSCO
Chrisman (2010) (66)	United States of America	To review the literature on current practices for managing MFWs in palliative and end-of-life patients, focusing on symptom management.	Narrative review	EBSCO, PubMed, Scopus, Web of Science
Clark (2002) (67)	United Kingdom	To review the literature on the use of metronidazole preparations to manage malodorous MFW.	Narrative review	EBSCO, PubMed, Scopus
Da Costa Santos et al. (2010) (65)	Brazil	To review the literature on topical treatment to manage the odor of MFWs.	Systematic review	EBSCO, PubMed, Scopus, Web of Science
De Oliveira Souza et al. (2018) (35)	Brazil	To review the literature on the instruments for the assessment of odor in MFWs.	Integrative review	EBSCO
Dowsett (2002) (68)	United Kingdom	To investigate the assessment of patients with MFWs in the community.	Narrative review	EBSCO, PubMed, Scopus
Dutta et al. (2022) (36)	Canada	To evaluate the changes in the degree of comfort before and after the treatment of MFWs.	Observational study	EBSCO, PubMed, Scopus, Web of Science
Firmino et al. (2020) (37)	Brazil	To describe the prevalence, characteristics, and associated factors of MFW in hospitalized patients.	Observational study	EBSCO
Firmino et al. (2020) (38)	Brazil	To evaluate the efficacy of regenerated oxidized cellulose compared to calcium alginate in managing the bleeding of MFWs (breast).	Randomized-controlled trial	EBSCO
Fromantin et al. (2014) (39)	France	To evaluate the use of various care procedures and the characteristics of MFWs.	Observational study	EBSCO
Furka et al. (2022) (40)	Hungary	To review the literature on the treatment options for managing MFWs.	Systematic review	EBSCO, PubMed, Scopus, Web of Science
Grocott (1997) (53)	United Kingdom	To evaluate the use of the TELER instrument in assessing the effectiveness of dressings used in patients with MFWs.	Case study	EBSCO, PubMed, Scopus
Grocott (1998) (54)	United Kingdom	To evaluate dressing performance in the management of exudating MFWs.	Case study	EBSCO, PubMed, Scopus
Grocott (2000) (60)	United Kingdom	To evaluate the efficacy of selected dressing materials in managing MFW symptoms.	Clinical trial	EBSCO, PubMed, Scopus, Web of Science
Grocott and Cowley (2001) (55)	United Kingdom	To focus on the methodological issues and the nature of the evidence generated by a case study on MFW.	Case study	EBSCO, PubMed, Scopus, Web of Science
Grocott (2001) (56)	United Kingdom	To evaluate how selected dressing materials manage MFW symptoms and introduce novel dressing systems.	Case study	EBSCO, Scopus
Hawthorn (2010) (57)	United Kingdom	To report on the issues of symptom management of MFWs and the implications for improving and maintaining the quality of care.	Case study	PubMed, Scopus
Kelechi et al. (2017) (41)	United States of America	To evaluate the effect of RGN107 topical wound powder in reducing pain, odor, and exudate in patients with MFWs.	Observational study	EBSCO, Scopus, Web of Science
Lai et al. (2003) (61)	Taiwan	To evaluate the combined efficacy of topical As203 and radiation therapy on MFWs in patients with breast cancer.	Clinical trial	PubMed, Scopus
Lian et al. (2014) (42)	Singapore	To evaluate the efficacy of green tea compared to conventional topical metronidazole powder in reducing the malodor of MFWs.	Randomized-controlled trial	EBSCO, Scopus
Lo et al. (2012) (58)	Taiwan	To describe the relationship between symptoms and quality of life in patients with MFWs.	Observational study	EBSCO, PubMed, Scopus, Web of Science
Lund-Nielsen et al. (2005) (62)	Denmark	To investigate the experience of women with advanced breast cancer and MFWs, evaluating the effects of a management regimen combined with psychosocial support.	Mixed-methods	EBSCO, PubMed, Scopus
Lund-Nielsen et al. (2011) (59)	Denmark	To evaluate the efficacy of honey-coated compared with silver-coated bandages on the treatment of MFWs.	Randomized-controlled trial	EBSCO
Naylor (2001) (69)	United Kingdom	To review the literature on the pain physiology of MFW.	Narrative review	EBSCO, PubMed, Scopus
Peng and Dai (2019) (43)	China	To evaluate the efficacy of metronidazole combined with autolytic debridement in managing the malodor of MFWs.	Randomized-controlled trial	Web of Science
Patel et al. (2018) (44)	India	To evaluate the effects of systemic triple therapy (ivermectin, albendazole, and clindamycin) in reducing signs and symptoms of MFWs.	Observational study	PubMed, Scopus
Ramasubbu et al. (2017) (45)	United Kingdom	To review the literature on the effects of systemic antibiotics on MFWs.	Systematic review	PubMed, Scopus, Web of Science
Savage et al. (2019) (46)	Canada	To validate the MWAT-R.	Validity study	EBSCO
Schulz et al. (2009) (64)	Canada	To validate the MWAT-C and the MWAT-R.	Validity study	EBSCO
Seaman (2006) (70)	United States of America	To review the literature on the pathophysiology, assessment, and symptom management in patients with MFWs.	Narrative review	EBSCO, PubMed, Scopus
Tamai et al. (2013) (63)	Japan	To describe the morphological characteristics of moisture-associated dermatitis and their related factors in patients with MFWs.	Mixed-methods	EBSCO, PubMed, Scopus, Web of Science
Tamai et al. (2016) (47)	Japan	To evaluate the intensity of pain and their association with wound status in patients with MFWs.	Observational study	EBSCO, PubMed, Scopus, Web of Science
Tang et al. (2020) (48)	China	To evaluate the effects of topical oxygen therapy on MFWs.	Case study	Web of Science
Watanabe et al. (2016) (49)	Japan	To evaluate the efficacy and safety of topical metronidazole gel in reducing the malodor of MFWs.	Clinical trial	EBSCO, PubMed, Scopus, Web of Science
Winardi and Irwan (2019) (50)	Indonesia	To review the literature on the use of topical treatments for managing the odor of MFWs.	Systematic review	EBSCO
Yasmara et al. (2024) (51)	Taiwan	To review the literature on the management of MFWs.	Scoping review	EBSCO
You et al. (2021) (52)	China	To describe the experience of caring for a patient with non-Hodgkin lymphoma and MFW.	Case study	EBSCO, PubMed, Scopus, Web of Science
Young (2005) (71)	United Kingdom	To investigate the transition from normal to altered body image in patients with MFWs and explore the nursing strategies for their assessment.	Narrative review	EBSCO, PubMed, Scopus

**Table 3:** Instruments and signs and symptoms evaluated.

Reference	Instrument	Signs and symptoms	Language
Adderley and Holt (2014) (32)	Visual Analogue Scale Verbal Rating Scale	Pain, Exudate, Odor	English
Agra et al. (2015) (33)	Numerical Rating Scale	Pain	Portuguese
Agra et al. (2019) (34)	Visual Analogue Scale	Pain	Portuguese
Chrisman (2010) (66)	Treatment Evaluation by Le Roux Wound Symptoms Self-Assessment Chart Visual Analogue Scale Faces Rating Scale Face Legs Cry and Consolability scale McGill Pain Questionnaire Pressure Ulcer Scale for Healing	Pain, Exudate	English
Clark (2002) (67)	Visual Analogue Scale	Odor	English
Da Costa Santos et al. (2010) (65)	Visual Analogue Scale	Odor	English
De Oliveira Souza et al. (2018) (35)	Treatment Evaluation by Le Roux Visual Analogue Scale Verbal Rating Scale	Odor	English Portuguese
Dowsett (2002) (68)	Visual Analogue Scale Verbal Rating Scale	Pain	English
Dutta et al. (2022) (36)	Edmonton Symptom Assessment System	Pain, Discomfort	English
Firmino et al. (2020) (37)	Visual Analogue Scale Verbal Rating Scale	Pain	English
Firmino et al. (2020) (38)	Hopkins Wound Assessment Tool Bleeding Intensity Scale	Wound stage, Bleeding	English
Fromantin et al. (2014) (39)	Verbal Rating Scale	Pain, Exudate	English
Furka et al. (2022) (40)	Treatment Evaluation by Le Roux	Odor	English
Grocott (1997) (53)	Treatment Evaluation by Le Roux	Exudate, Dressing change, Dressing fit	English
Grocott (1998) (54)	Treatment Evaluation by Le Roux	Exudate, Dressing change, Dressing fit	English
Grocott (2000) (60)	Treatment Evaluation by Le Roux	Exudate, Dressing fit, Irritation, Erythema	English
Grocott and Cowley (2001) (55)	Treatment Evaluation by Le Roux	Exudate, Dressing change, Dressing fit	English
Grocott (2001) (56)	Treatment Evaluation by Le Roux	Exudate, Dressing fit, Discomfort, Skin condition, Odor	English
Hawthorn (2010) (57)	Edmonton Symptom Assessment System	Pain, Fatigue, Anxiety, Insomnia	English
Kelechi et al. (2017) (41)	Visual Analogue Scale Pain Assessment In Advanced Dementia McGill Pain Questionnaire	Pain, Exudate, Odor	English
Lai et al. (2003) (61)	Visual Analogue Scale	Pain	English
Lian et al. (2014) (42)	Verbal Numeric Scale	Odor	English
Lo et al. (2012) (58)	Malignant Wound Assessment Tool – Perception Malignant Wound Assessment Tool – wound bed status	Pain, Odor, Bleeding, Exudate, Edema, Wound Area, Psychological Issue, Social Issue, Tissue, Dressing comfort	English
Lund-Nielsen et al. (2005) (62)	Verbal Rating Scale	Odor, Bleeding, Exudate	English
Lund-Nielsen et al. (2011) (59)	Visual Analogue Scale Verbal Rating Scale	Odor, Exudate, Pain	English
Naylor (2001) (69)	Visual Analogue Scale Faces Rating Scale Verbal Numeric Scale Verbal Rating Scale	Pain	English
Peng and Dai (2019) (43)	Treatment Evaluation by Le Roux	Odor	English
Patel et al. (2018) (44)	Wound Assessment Tool hospice Edmonton Symptom Assessment System	Pain, Odor, Exudate, Itching, Bleeding, Edema, Distress, Maggots	English
Ramasubbu et al. (2017) (45)	Treatment Evaluation by Le Roux Visual Analogue Scale Verbal Numeric Scale	Odor, Pain	English
Savage et al. (2019) (46)	Malignant Wound Assessment Tool – Research	Pain, Odor, Drainage, Bleeding, Skin change, Swelling, Social issues	English
Schulz et al. (2009) (64)	Malignant Wound Assessment Tool – Clinical Malignant Wound Assessment Tool – Research	Pain, Odor, Drainage, Bleeding, Skin change, Exudate, Social issues, Wound size	English
Seaman (2006) (70)	Hopkins Wound Assessment Tool Visual Analogue Scale	Wound color, Hydration status, Presence of nodules, Drainage, Pain, Odor, Tunneling	English
Tamai et al. (2013) (63)	Bates-Jensen Wound Assessment Tool Malignant Wound Assessment Tool – Research	Size, Depth, Necrotic tissue, Exudate, Skin, Edema, Granulation	English
Tamai et al. (2016) (47)	Short-form McGill Pain Questionnaire Bates-Jensen Wound Assessment Tool	Pain, Size, Depth, Edges, Undermining, Necrotic tissue, Exudate, Skin color, Edema, Induration, Granulation, Epithelization	English
Tang et al. (2020) (48)	Visual Analogue Scale	Pain, Odor	English
Watanabe et al. (2016) (49)	Visual Analogue Scale	Pain, Odor, Dressing	English
Winardi and Irwan (2019) (50)	Verbal Rating Scale	Odor	English
Yasmara et al. (2024) (51)	Toronto Symptom Assessment System for Wound Malignant Wound Assessment Tool – Clinical Malignant Wound Assessment Tool – Research	Pain, Odor, Drainage, Bleeding, Skin change, Exudate, Social issues, Wound Size	English
You et al. (2021) (52)	Treatment Evaluation by Le Roux	Odor	English
Young (2005) (71)	Treatment Evaluation by Le Roux	Odor, Exudate, Dressing, Pain	English

**Figure 2:**
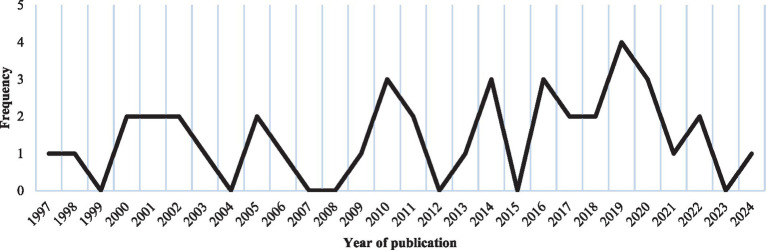
Number of included records per year of publication.

The publication period of the included studies ranges from 1997 to 2024. The distribution of studies over these years is heterogeneous, with a marked increase in research interest over the past decade (*n* = 21, 52.5%) (32–52). The highest number of eligible studies was published in 2019 (34, 43, 46, 50), as illustrated in Figure 2.

In terms of study design, most of the studies included were primary research (*n* = 27, 67.5%), comprising case studies (*n* = 8, 20%) (33, 48, 52–57), observational studies (*n* = 8, 20%) (34, 36, 37, 39, 41, 44, 47, 58), randomized controlled trials (*n* = 4, 10%) (38, 42, 43, 59), clinical trials (*n* = 3, 7.5%) (49, 60, 61), mixed-methods research (*n* = 2, 5%) (62, 63), and validation studies (*n* = 2, 5%) (46, 64). Secondary research was less common (*n* = 13, 32.5%), consisting primarily of systematic reviews (*n* = 5, 12.5%) (32, 40, 45, 50, 65) and other literature reviews (*n* = 8, 20%) (35, 51, 66–71).

**Figure 3:**
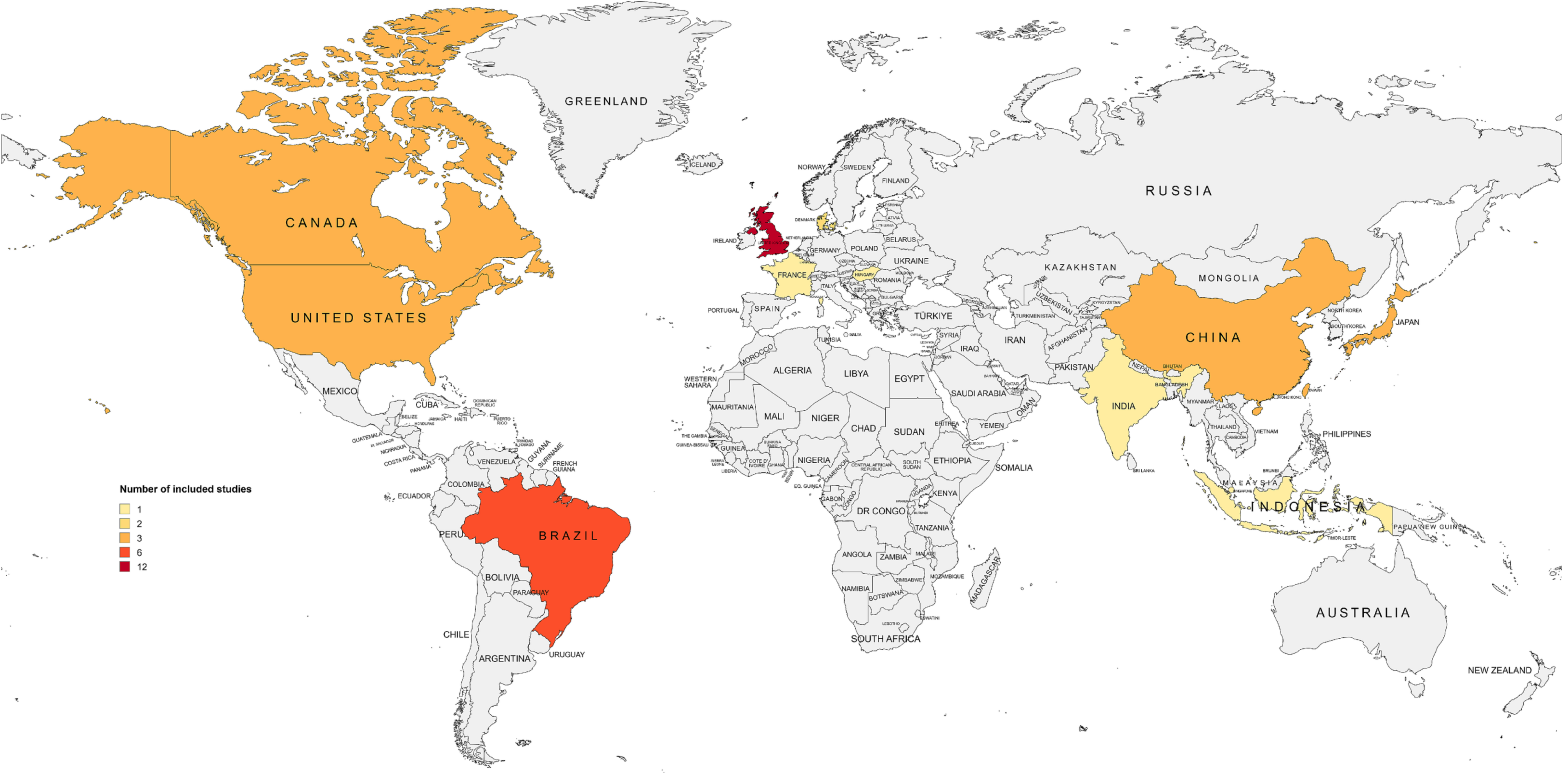
Map of included records per country.

Geographically, the included studies originated from 13 countries in six macro-regions: Europe (*n* = 16, 40%) (32, 39, 40, 45, 53–57, 59, 60, 62, 67–69, 71), East Asia (*n* = 9, 22.5%) (43, 47–49, 51, 52, 58, 61, 63), North America (*n* = 6, 15%) (36, 41, 46, 64, 66, 70), South America (*n* = 6, 15%) (33–35, 37, 38, 65), the Pacific (*n* = 2, 5%) (42, 50), and South Asia (*n* = 1, 2.5%) (44). As shown in Figure 3, the United Kingdom contributed the largest number of eligible studies (*n* = 12, 30%) (32, 45, 53–57, 60, 67–69, 71). Most of the included articles were published in English (*n* = 37, 92.5%) (32, 36–46, 53–62, 64–70), followed by Portuguese (*n* = 2, 5%) (33, 34), while one record (*n* = 1, 2.5%) (35) appeared in both languages.

**Figure 4:**
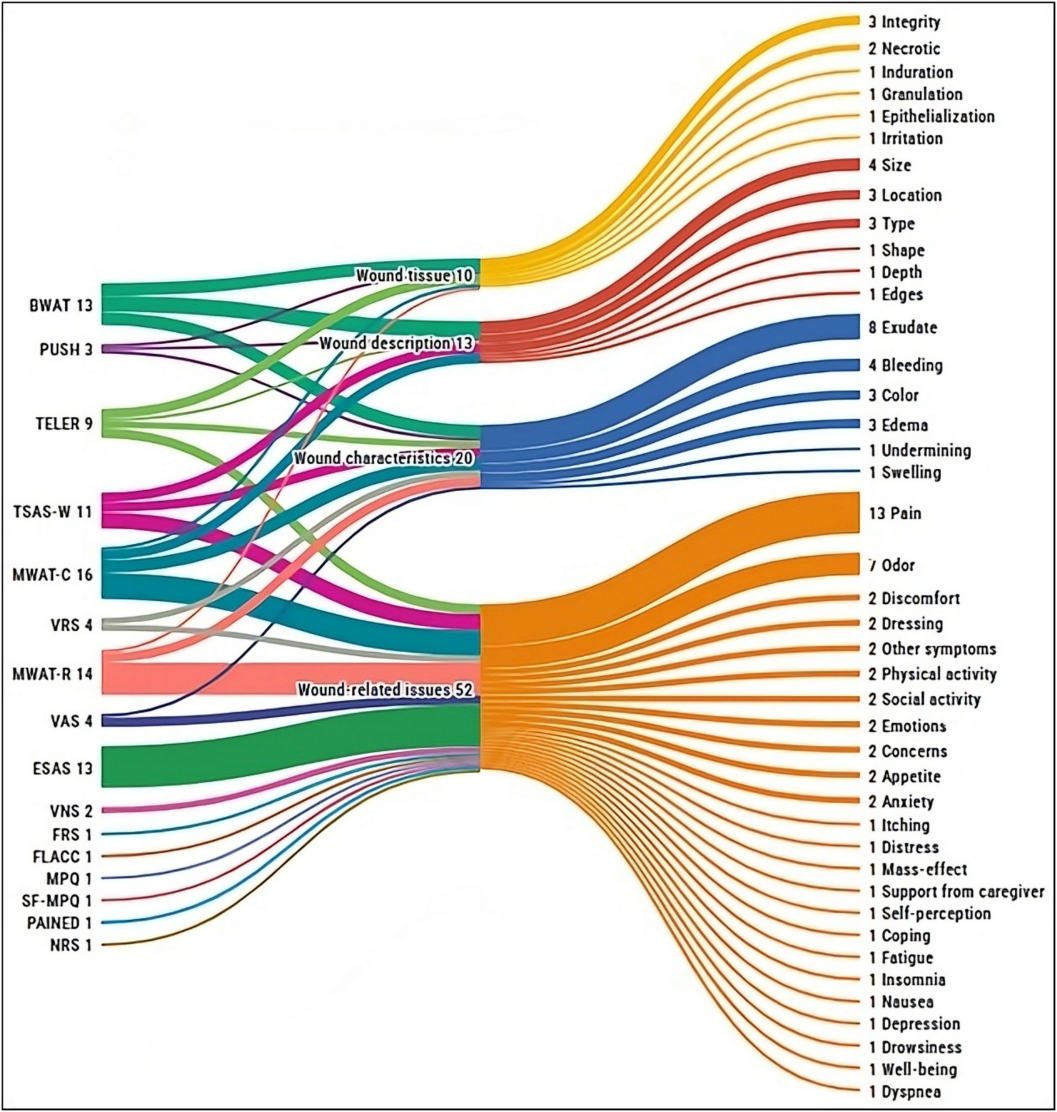
Overview of signs and symptoms assessed by the validated instruments, grouped by instrument and dimension evaluated. BWAT, Bates-Jensen Wound Assessment Tool; ESAS, Edmonton Symptom Assessment System; FLACC, Face Legs Cry and Consolability Scale; FRS, Faces Rating Scale; MPQ, McGill Pain Questionnaire; MWAT-C, Malignant Wound Assessment Tool – Clinical; MWAT-R, Malignant Wound Assessment Tool – Research; NRS, Numerical Rating Scale; PAINAD, Pain Assessment In Advanced Dementia; PUSH, Pressure Ulcer Scale for Healing; SF-MPQ, Short-form McGill Pain Questionnaire; TELER, Treatment Evaluation by Le Roux; TSAS-W, Toronto Symptom Assessment System for Wound; VAS, Visual Analogue Scale; VNS, Verbal Numeric Scale; VRS, Verbal Rating Scale.

Finally, 22 distinct instruments were identified across the included studies (Tables 2, 3). Out of these, 13 studies (32.5%) reported on two or more tools concurrently, resulting in a total of 68 instances of tool reporting. The Visual Analogue Scale (*n* = 16, 23.5%) and the Treatment Evaluation by Le Roux (*n* = 12, 17.6%) emerged as the most cited. Half of the instruments (*n* = 11) were specifically developed to assess wounds and related signs and symptoms, whereas the other half (*n* = 11) targeted general symptoms such as pain or insomnia, without a wound-specific focus. Furthermore, considering their validity, most of the assessment tools identified were previously validated (*n* = 16, 72.7%), whereas a minority lacked formal validity testing (*n* = 6, 27.3%). Lastly, as illustrated in Figure 4, the most common signs and symptoms evaluated by validated instruments were pain (*n* = 12, 78.5%), exudate (*n* = 8, 57.1%), and odor (*n* = 7, 50%). The following sections discuss each tool and its investigated dimensions, signs, and symptoms in detail, distinguishing between wound-specific and non-specific instruments.

### Wound-specific instruments for sign and symptom assessment

Of the tools employed to assess MFWs’ signs and symptoms, most (*n* = 7, 66.6%) were originally developed for other wound types (e.g., pressure ulcers) and subsequently applied to MFWs. These include the Bates-Jensen Wound Assessment Tool (BWAT), the Hopkins Wound Assessment Tool (HWAT), the Pressure Ulcer Scale for Healing (PUSH), the Treatment Evaluation by Le Roux (TELER), the Toronto Symptom Assessment System for Wound (TSAS-W), the Wound Assessment Tool for hospices (WAT), and the Wound Symptoms Self-Assessment Chart (WoSSAC). A smaller subset (*n* = 4, 36.3%) was specifically designed for MFWs, comprising four versions of the Malignant Wound Assessment Tool (MWAT): Clinical (MWAT-C), Research (MWAT-R), Wound Bed Status (MWAT-N), and Perception (MWAT-P).

**Figure 5:**
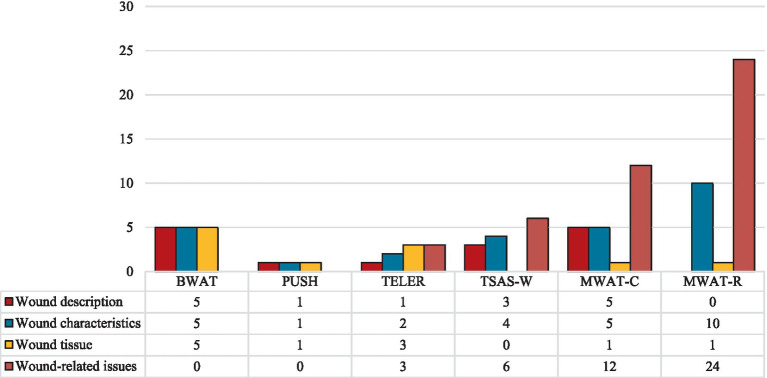
Signs and symptoms assessed by the validated wound-specific instruments, grouped by instrument and dimension evaluated. BWAT, Bates-Jensen Wound Assessment Tool; MWAT-C, Malignant Wound Assessment Tool – Clinical; MWAT-R, Malignant Wound Assessment Tool – Research; PUSH, Pressure Ulcer Scale for Healing; TELER, Treatment Evaluation by Le Roux; TSAS-W, Toronto Symptom Assessment System for Wound.

Figure 5 illustrates the dimensions covered by each validated wound-specific instrument, along with the percentage of items that address each dimension. Overall, the MWAT-R includes the broadest range of wound-related items (*n* = 24), capturing aspects such as pain and odor alongside their impacts on social interactions, physical activity, appetite, self-perception, and emotional well-being. MWAT-C also addresses various wound-related issues (*n* = 12), further detailing wound characteristics such as location, size, changes over time, exudate, bleeding, and edema.

### Non-specific instruments for sign and symptom assessment

**Figure 6:**
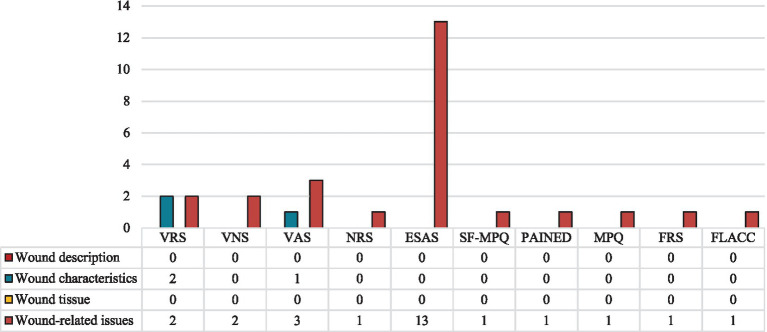
Signs and symptoms assessed by the validated non-specific instruments, grouped by instrument and dimension assessed. ESAS, Edmonton Symptom Assessment System; FLACC, Face Legs Cry and Consolability scale; FRS, Faces Rating Scale; MPQ, McGill Pain Questionnaire; NRS, Numerical Rating Scale; PAINAD, Pain Assessment In Advanced Dementia; SF-MPQ, Short-form McGill Pain Questionnaire; VAS, Visual Analogue Scale; VNS, Verbal Numeric Scale; VRS, Verbal Rating Scale.

Most non-wound-specific tools identified (*n* = 6, 54.5%) focus on evaluating a single symptom. These include the Bleeding Intensity Scale (BIS); Face, Legs, Activity, Cry, and Consolability (FLACC) scale; Faces Rating Scale (FRS); McGill Pain Questionnaire (MPQ); Pain Assessment in Advanced Dementia (PAINAD); and Short-Form McGill Pain Questionnaire (SF-MPQ). The remaining instruments (*n* = 5, 45.5%) assess multiple symptoms, such as the Edmonton Symptom Assessment System (ESAS) and widely used, simple scales like the Numerical Rating Scale (NRS), Visual Analogue Scale (VAS), Verbal Numeric Scale (VNS), and Verbal Rating Scale (VRS). These tools are valued for their feasibility and Likert-like structure, making them adaptable for various clinical contexts. Almost all (*n* = 10, 90.9%) have been validated in English (72–77), except the BIS. As shown in Figure 6, none of these instruments address wound characteristics or tissue integrity, but all capture at least one wound-related symptom, such as pain. Only two were also employed to describe wound characteristics: the VAS for exudate amount and the VRS for both exudate and bleeding.

## Discussion

This scoping review identified the instruments used for MFWs’ assessment, which remain a significant clinical challenge in patients with advanced cancer. MFWs have a negative impact on psycho-social and emotional quality of life (78), affecting body image perception and contributing to worse distress (79), due to related symptoms, such as pain, excessive exudate, malodor, and bleeding (1). Malodor is particularly distressing, often contributing to stigma and social withdrawal, making patients reluctant to seek medical attention (10). The visible and odorous nature of MFWs can also act as a distressing reminder of disease progression and the imminence of death, exacerbating anxiety for both patients and their families (80). Stigma or fear, especially when wounds occur in sensitive areas such as the breast, face, or genitals, further discourages patients from seeking timely care (81). Given these challenges, a comprehensive and multidimensional assessment of MFWs is crucial (46), although these wounds do not have a good healing prognosis. Our results described multiple tools that have been used to assess MFWs, which most capture only a limited subset of wound-related signs and symptoms. Therefore, this review partially addresses the research question, as the available instruments, while some are specific to the assessment of MFWs, cannot achieve a comprehensive and multidimensional evaluation. Half of the instruments were specifically developed to assess wounds, and related signs and symptoms (i.e., bleeding, odor, exudate), and the other half targeted general symptoms such as pain, anxiety, and insomnia, without a wound-specific focus. Our research identified the MWAT in four different versions, but only the MWAT-C and MWAT-R (64) are specifically for assessing and managing MFWs. The MWAT-C addresses several wound-related issues, particularly wound characteristics such as location, size, changes over time, exudate, bleeding, and edema. Instead, the MWAT-R includes a broad range of wound-related items, capturing clinical characteristics of the wound and social interactions, physical activity, self-perception, and emotional well-being, and also focuses on nutritional and self-esteem. A systematic approach is optimal for the evaluation of patients presenting with MFWs, given the infrequent etiology of a singular underlying cause. A comprehensive assessment of both local and systemic contributing factors within each stage of the diagnostic work-up is critical. The principles guiding ongoing nursing and clinician assessment and monitoring of wounds generally exhibit considerable overlap (82). It is plausible that managing skin wounds with bad healing prognoses does not solicit standardized clinical and welfare responses, with careful funds to support research (83). All health professionals, and in particular nurses, are now part of the healthcare team as patient advocates. They must respond to health needs as a nursing imperative, supporting public health as a fundamental right (84).

A key insight of this review is the lack of standardized, multidimensional instruments capable of capturing both physical and psychosocial domains of MFWs with a single tool. The MWAT-C and MWAT-R strive toward such comprehensiveness by integrating wound characteristics, social impacts, and emotional dimensions. Moreover, they fail to address certain symptoms frequently reported in the literature, such as itching and the presence of maggots (22). Although these symptoms can be recorded under an open-ended other symptoms category, this approach relies on practitioners’ awareness and assessment skills, potentially introducing variability and inconsistency in evaluations. As our review indicates, healthcare professionals often use separate instruments to evaluate different aspects, such as pain intensity, exudate volume, or odor severity, leading to fragmented assessments and repeated measurements. This fragmentation not only places an additional burden on patients but can also reduce adherence to regular assessments (20). Reflecting best practice recommendations (grade B) and recognized as a quality indicator for the physical aspects of palliative care, nurses and other healthcare professionals should utilize evidence-based assessment tools for the initial and ongoing evaluation of symptoms in end-of-life care patients (84, 85). As highlighted by the guidelines generated by the European Oncology Nursing Society (EONS) only some scales assess psychosocial aspects and their impact on daily life; therefore having an integrated and comprehensive instrument for the assessment of MFWs is vital for guiding effective palliative strategies, particularly in situations where patients are facing profound physical, emotional, and social strains. A comprehensive assessment should take into account the physical and psychosocial aspects of both the patient and caregivers, treatment such as symptoms evaluation, supportive care, and preventative measures, and a focus on local wound management such as bleeding, odor, pain/pruritus, exudate, and superficial infection (86). The substantial metabolic demands of patients with MFWs, often due to significant wound exudate or fistula fluid, necessitate a higher energy intake. Standard guidelines recommend 25–35 kcal/kg body weight per day, so frequent meals and snacks may be indicated for individuals with MFWs to satisfy these elevated energy requirements (87). The National Health Service Forth Valley guidelines for the management of MFWs suggest the M: EMPHIS Pathway approach which is specific for guiding the management of local symptoms about malignant wound (M), exudate (E), malodour (M), pain (P), hemorrhage (H), infection (I) and skin tissue related problems (S) (88). This may be a good approach to manage the core symptoms related to MFWs, but it does not address the other symptoms that occur in patients with this lesion. Nurses report difficulties in applying wound dressings and concealing their disgust at the odor, often finding the care of these patients emotionally challenging due to the sometimes incurable nature of the wounds, while also facing the complexities of patient isolation, altered body image, and holistic interdisciplinary approach guided by specialist-level palliative wound care expertise (22). Effectively managing the complex needs of patients with MFWs demands a pragmatic approach. Implementing palliative care relies on a holistic approach that spans multiple levels of assistance, involving the collaborative input of various disciplines from the health, education, business, and labor sectors, all guided by a transdisciplinary framework to address these needs. It’s vital to consider the transdisciplinary nature of palliative care, enabling diverse professions to contribute their specific knowledge toward a common objective, which includes integrating family and community. Their experiences and inherited knowledge can significantly inform the goal of addressing the needs of the individual-family dyad (89). Interdisciplinary care involves assistance where knowledge is shared at the point of care delivery and where the goal of care is agreed upon. There is communication, collaboration, and coordination among the various care providers. The importance of each discipline and the contribution each can make is recognized to deliver integrated and individualized palliative care based on the needs of the patient-family dyad (90).

Comprehensive and easy-to-use tools, and an interdisciplinary team could facilitate the use of a standardized language, help nurses and healthcare professionals to effectively assessment the complexities of MFWs, reduce wound management times, ensure the assessment of all the symptoms, and provide well-being in the patient-family dyad (91). Therefore, it is imperative to investigate the adoption and implementation of efficient tools informed by scientific evidence, which support the transition toward more efficacious care paradigms, as exemplified by transdisciplinary palliative care.

Additionally, the MWAT-C and the MWAT-R have only been validated in English and lack comprehensive psychometric testing, raising concerns about their reliability and limiting their applicability in diverse cultural contexts (92). Validation in terms of both validity and reliability is essential to ensure that an instrument accurately measures the intended constructs and can be confidently applied across different clinical and research settings. Without such validation, the use of these instruments risks introducing measurement errors, leaving practices prone to error in contexts not oriented toward evidence-based practice (93).

### Strengths and limitations

The findings of this review should be interpreted in light of a few limitations. First, the search was limited to four databases, which may have resulted in the omission of relevant studies indexed elsewhere. Additionally, to ensure a timely completion while upholding methodological rigor, gray literature was not included. This may have narrowed the scope of the findings and excluded potentially valuable insights. Furthermore, the limited validation of assessment tools across different languages and cultural contexts may hinder the global applicability of current approaches to the evaluation of MFWs. Finally, the sensitive nature of the topic, where patients may hide their wounds due to stigma or embarrassment, could lead to underreporting thereby affecting the generalizability of existing evidence, and could be a potential bias (10).

## Conclusion

The assessment of MFWs continue to raise significant challenges due to the lack of standardized, comprehensive, and evidence-based evaluation tools. Although various instruments have been employed, most focus only on specific dimensions of MFWs, leading to fragmented assessments and potentially suboptimal care. Among the available measures, the MWAT-C and MWAT-R demonstrate the greatest potential, due to their relatively broad scope. However, both tools still require further refinement and validation, especially to incorporate underreported symptoms and enhance applicability across diverse clinical and cultural settings. There is an urgent need for the development of a validated, multidimensional instrument specifically designed to capture the complexity of MFWs within a broader biopsychosocial framework. In the interim, in the absence of such tools, nurses are encouraged to follow evidence-based and adapt clinical guidelines in different clinical context (94), such as those from the EONS and NHS (86, 88), to guide wound assessment in clinical practice. Future research should prioritize rigorous investigations that address existing gaps in knowledge and practice, moving beyond traditional, non-standardized approaches. This process will ultimately improve the assessment of MFWs in palliative care settings and provide better support to patients, their families, and healthcare professionals.

## References

[1] Starace M Carpanese MA Pampaloni F Dika E Pileri A Rubino D. Management of malignant cutaneous wounds in oncologic patients. Support Care Cancer. (2022) 30:7615–23. doi: 10.1007/s00520-022-07194-0 35672478 PMC9385755

[2] Mohan S Khan A. Pain and wound management in fungating Merkel cell carcinoma within a palliative setting: the first case report of this predicament. Indian J Palliat Care. (2024) 30:81–4. doi: 10.25259/IJPC_259_2023 38633689 PMC11021067

[3] Hasan N Nadaf A Imran M Jiba U Sheikh A Almalki WH. Skin cancer: understanding the journey of transformation from conventional to advanced treatment approaches. Mol Cancer. (2023) 22:168. doi: 10.1186/s12943-023-01854-3 37803407 PMC10559482

[4] Adderley U Smith R. Topical agents and dressings for fungating wounds In: The Cochrane Collaboration, editor. The Cochrane database of systematic reviews (protocol). Chichester, UK: John Wiley & Sons, Ltd. (2003). CD003948. 10.1002/14651858.CD003948.pub2 17443534

[5] O’Neill L Nelson Z Ahmad N Fisher AH Denton A Renzi M. Malignant fungating wounds of the head and neck: management and antibiotic stewardship. OTO Open. (2022) 6:3306. doi: 10.1177/2473974X211073306 PMC8832587 35155974

[6] Tsichlakidou A Govina O Vasilopoulos G Kavga A Vastardi M Kalemikerakis I. Intervention for symptom management in patients with malignant fungating wounds – a systematic review. J BUON. (2019) 24:1301–8. 31424694

[7] Alexander S. Malignant fungating wounds: epidemiology, aetiology, presentation and assessment. J Wound Care. (2009) 18:273–80. doi: 10.12968/jowc.2009.18.7.43110 19827480

[8] Gethin G Vellinga A McIntosh C Sezgin D Probst S Murphy L. Systematic review of topical interventions for the management of odour in patients with chronic or malignant fungating wounds. J Tissue Viability. (2023) 32:151–7. doi: 10.1016/j.jtv.2022.10.007 36376189

[9] Morris C. Wound odour: principles of management and the use of Clini sorb. Br J Nurs. (2008) 17:S38–42. doi: 10.12968/bjon.2008.17.Sup3.28914 18524023

[10] Dos Santos WA Fuly PDSC Souto MD Dos Santos MLSC Beretta LDL. Asociación entre olor y aislamiento social en pacientes con heridas tumorales malignas: estudio piloto. Eglobal. (2018) 18:19–65. doi: 10.6018/eglobal.18.1.322641

[11] Koumaki D Kostakis G Boumpoucheropoulos S Ioannou P Katoulis AC. A narrative review of management of wounds in palliative care setting. Ann Palliat Med. (2023) 12:1089–105. doi: 10.21037/apm-23-138 37859426

[12] Liu X Xie JQ Liao ZY Wei MJ Lin H. Changes in wound symptoms and quality of life of patients with newly diagnosed malignant fungating wounds. J Wound Care. (2024) 33:262–70. doi: 10.12968/jowc.2024.33.4.262, PMID: 38573899

[13] Tam SH Lai WS Kao CY Fang SY. Maintain professionalism: nurses’ experiences in caring for patients with malignant Fungating wounds in Taiwan. J Pain Symptom Manag. (2024) 68:69–77.e1. doi: 10.1016/j.jpainsymman.2024.04.008 38621610

[14] Huang Y Hu J Xie T Jiang Z Ding W Mao B. Effects of home-based chronic wound care training for patients and caregivers: a systematic review. Int Wound J. (2023) 20:3802–20. doi: 10.1111/iwj.14219 37277908 PMC10588341

[15] Eyres J. Fear of malignant fungating wounds. Br J Community Nurs. (2024) 29:S36–41. doi: 10.12968/bjcn.2024.29.Sup9.S36 39240812

[16] Alexander SJ. An intense and unforgettable experience: the lived experience of malignant wounds from the perspectives of patients, caregivers and nurses. Int Wound J. (2010) 7:456–65. doi: 10.1111/j.1742-481X.2010.00715.x 20673255 PMC7951249

[17] Cornish L. Holistic management of malignant wounds in palliative patients. Br J Community Nurs. (2019) 24:S19–23. doi: 10.12968/bjcn.2019.24.Sup9.S19 31479334

[18] Atthayasai J Chatchumni M Eriksson H Mazaheri M. Surgical nurses' perceptions of strategies to enhance pain management proficiency: a qualitative study. Nurs Rep. (2023) 13:923–33. doi: 10.3390/nursrep13020081, PMID: 37368348 PMC10301761

[19] Ferrell B Virani R Malloy P Kelly K. The preparation of oncology nurses in palliative care. Semin Oncol Nurs. (2010) 26:259–65. doi: 10.1016/j.soncn.2010.08.001, PMID: 20971406

[20] Schmidt FMQ Firmino F Lenza NDFB Santos VLCDG. Nursing team knowledge on care for patients with fungating wounds. Rev Bras Enferm. (2020) 73:e20170738. doi: 10.1590/0034-7167-2017-0738, PMID: 32049214

[21] Probst S Arber A Faithfull S. Malignant fungating wounds: a survey of nurses’ clinical practice in Switzerland. Eur J Oncol Nurs. (2009) 13:295–8. doi: 10.1016/j.ejon.2009.03.008 19386546

[22] Tilley CP Fu MR Van Cleeve J Crocilla BL Comfort CP. Symptoms of malignant fungating wounds and functional performance among patients with advanced cancer: an integrative review from 2000 to 2019. J Palliat Med. (2020) 23:848–62. doi: 10.1089/jpm.2019.0617 32349622

[23] Chang SL Chung CF Liou YG Lo SF Hu SH. Improving malignant fungating wound management among oncology nurses: a best practice implementation project. JBI Evid Implement. (2025) 23:33–41. doi: 10.1097/XEB.0000000000000430 38738475

[24] Tilley C Lipson J Ramos M. Palliative wound care for malignant fungating wounds. Nurs Clin North Am. (2016) 51:513–31. doi: 10.1016/j.cnur.2016.05.006 27497023

[25] Wilkes LM Boxer E White K. The hidden side of nursing: why caring for patients with malignant malodorous wounds is so difficult. J Wound Care. (2003) 12:76–80. doi: 10.12968/jowc.2003.12.2.26468 12655971

[26] Arksey H O’Malley L. Scoping studies: towards a methodological framework. Int J Soc Res Methodol. (2005) 8:19–32. doi: 10.1080/1364557032000119616

[27] Peters MDJ Marnie C Tricco AC Pollock D Munn Z Alexander L. Updated methodological guidance for the conduct of scoping reviews. JBI Evid Synth. (2020) 18:2119–26. doi: 10.11124/JBIES-20-00167, PMID: 33038124

[28] Pollock D Davies EL Peters MDJ Tricco AC Alexander L McInerney P. Undertaking a scoping review: a practical guide for nursing and midwifery students, clinicians, researchers, and academics. J Adv Nurs. (2021) 77:2102–13. doi: 10.1111/jan.14743 33543511 PMC8049063

[29] Tricco AC Lillie E Zarin W O’Brien KK Colquhoun H Levac D. PRISMA extension for scoping reviews (PRISMA-ScR): checklist and explanation. Ann Intern Med. (2018) 169:467–73. doi: 10.7326/M18-0850 30178033

[30] Anastasi G Bambi S. Utilization and effects of security technologies in mental health: a scoping review. Int J Ment Health Nurs. (2023) 32:1561–82. doi: 10.1111/inm.13193, PMID: 37449535

[31] Page MJ McKenzie JE Bossuyt PM Boutron I Hoffmann TC Mulrow CD. Statement: an updated guideline for reporting systematic reviews. BMJ. (2020) 372:n71. doi: 10.1136/bmj.n71, PMID: 33782057 PMC8005924

[32] Adderley UJ Holt IGS. Topical agents and dressings for fungating wounds. Cochrane Database Syst Rev. (2014) 2014:CD003948. doi: 10.1002/14651858.CD003948.pub3, PMID: 24832784 PMC6464725

[33] Agra G de Lourdes André Gouveia B Tamar Oliveira Sousa A Lopes Costa MM Santos Oliveira SH Guimarães Soares MJ. Cuidados paliativos de enfermagem a paciente com carcinoma basocelular terebrante: estudo de caso. J Nurs UFPE. (2015) 9:9873–81.

[34] Agra G de Souza Medeiros MV Freire de Brito DT Silva Pimentel ER Soares Formiga N Lopes Costa MM. Conhecimento e prática de enfermeiros no controle da dor de pacientes com feridas neoplásicas. Enferm Bras. (2019) 18:3–11. doi: 10.33233/eb.v18i1.1039

[35] de Oliveira Souza MA Rodrigues de Souza N da Silva T Melo J Campos Absalão Xavier MA Lopes de Almeida G. Odor evaluation scales for odor in neoplastic wounds: an integrative review. Rev Bras Enferm. (2018) 71:2552–60. doi: 10.1590/0034-7167-2017-0428 30304189

[36] Dutta S Ishore K Ghoshal A. Role of integrative oncology and palliative care services in improving comfort level and compliance among patients with advanced fungating breast cancer—experience from a rural hospital of north eastern India during the COVID-19 pandemic. Indian J Palliat Care. (2022) 28:256–61. doi: 10.25259/IJPC_40_2021 36072251 PMC9443120

[37] Firmino F Ferreira SADC Franck EM De Queiroz WMS Castro DV Nogueira PC. Malignant wounds in hospitalized oncology patients: prevalence, characteristics, and associated factors. Plast Surg Nurs. (2020) 40:138–44. doi: 10.1097/PSN.0000000000000320, PMID: 32852440

[38] Firmino F Santos J Meira KC de Araújo JL Júnior VA de Gouveia Santos VLC. Regenerated oxidized cellulose versus calcium alginate in controlling bleeding from malignant breast cancer wounds: randomised control trial study protocol. J Wound Care. (2020) 29:52–60. doi: 10.12968/jowc.2020.29.1.52, PMID: 31930944

[39] Fromantin I Watson S Baffie A Rivat A Falcou MC Kriegel I. A prospective, descriptive cohort study of malignant wound characteristics and wound care strategies in patients with breast cancer. Ostomy Wound Manage. (2014) 60:38–48. 24905356

[40] Furka A Simkó C Kostyál L Szabó I Valikovics A Fekete G. Treatment algorithm for cancerous wounds: a systematic review. Cancers. (2022) 14:1177. doi: 10.3390/cancers14051177 35267512 PMC8909326

[41] Kelechi T Prentice M Madisetti M Brunette G Mueller M. Palliative care in the management of pain, odor, and exudate in chronic wounds at the end of life: a cohort study. J Hosp Palliat Nurs. (2017) 19:17–25. doi: 10.1097/NJH.0000000000000306

[42] Lian SB Xu Y Goh SL Aw FC. Comparing the effectiveness of green tea versus topical metronidazole powder in malodorous control of fungating malignant wounds in a controlled randomised study. Proc Singapore Healthc. (2014) 23:3–12. doi: 10.1177/201010581402300102

[43] Peng L Dai Y. Effect of metronidazole combined with autolytic debridement for the management of malignant wound malodor. J Int Med Res. (2019) 48. doi: 10.1177/0300060519897505 PMC7607146 31885300

[44] Patel BC Ostwal S Sanghavi PR Joshi G Singh R. Management of malignant wound myiasis with ivermectin, albendazole, and clindamycin (triple therapy) in advanced head-and-neck cancer patients: a prospective observational study. Indian J Palliat Care. (2018) 24:459–64. doi: 10.4103/IJPC.IJPC_92_18 30410258 PMC6199823

[45] Ramasubbu DA Smith V Hayden F Cronin P. Systemic antibiotics for treating malignant wounds. Cochrane Database Syst Rev. (2017) 8:CD011609. doi: 10.1002/14651858.CD011609.pub2, PMID: 28837757 PMC6483739

[46] Savage P Murphy-Kane P Lee CT Suet-Lam Chung C Howell D. Validation of the malignant wound assessment tool—research (MWAT-R) using cognitive interviewing. Can Oncol Nurs J. (2019) 29:97–109. doi: 10.5737/2368807629297102 31148749 PMC6516337

[47] Tamai N Mugita Y Ikeda M Sanada H. The relationship between malignant wound status and pain in breast cancer patients. Eur J Oncol Nurs. (2016) 24:8–12. doi: 10.1016/j.ejon.2016.05.004 27697281

[48] Tang J Qin F Chen Y Liu Y Nong L Zhong W. Topical oxygen therapy can reduce related symptoms of malignant fungating wounds in breast cancer: a retrospective observational case series study. Int J Clin Exp Med. (2020) 13:8526–34.

[49] Watanabe K Shimo A Tsugawa K Tokuda Y Yamauchi H Miyai E. Safe and effective deodorization of malodorous fungating tumors using topical metronidazole 0.75% gel (GK567): a multicenter, open-label, phase III study (RDT.07.SRE.27013). Support Care Cancer. (2016) 24:2583–90. doi: 10.1007/s00520-015-3067-0 26715293 PMC4846704

[50] Winardi A Irwan AM. Topical treatment for controlling malignant wound odour. EWMA J. (2019) 20:7–17. doi: 10.35279/jewma201910.01

[51] Yasmara D Tam SH Fang SY. Caring for patients with malignant fungating wounds: a scoping literature review. J Wound Ostomy Continence Nurs. (2024) 51:19–25. doi: 10.1097/WON.0000000000001046 38215293

[52] You M Zhang S Ma X Liu H Lu Y Li Y. Nursing of a non-Hodgkin’s lymphoma patient with a facial malignant fungating wound. Asia Pac J Oncol Nurs. (2021) 8:581–5. doi: 10.4103/apjon.apjon-2119 34527789 PMC8420912

[53] Grocott P. Evaluation of a tool used to assess the management of fungating wounds. J Wound Care. (1997) 6:421–4. doi: 10.12968/jowc.1997.6.9.421, PMID: 9370588

[54] Grocott P. Exudate management in fungating wounds. J Wound Care. (1998) 7:445–8. doi: 10.12968/jowc.1998.7.9.445, PMID: 9887735

[55] Grocott P Cowley S. The palliative management of fungating malignant wounds—generalising from multiple-case study data using a system of reasoning. Int J Nurs Stud. (2001) 38:533–45. doi: 10.1016/s0020-7489(00)00098-5 11524100

[56] Grocott P. Developing a tool for researching fungating wounds. World Wide Wounds [Internet]. 2001; 2001. Available online at: https://www.scopus.com/inward/record.uri?eid=2-s2.0–4043100470&partnerID=40&md5=59d582d5c0d7eebe4b1f51a8e140d020 (Accessed February 16, 2025).

[57] Hawthorn M. Caring for a patient with a fungating malignant lesion in a hospice setting: reflecting on practice. Int J Palliat Nurs. (2010) 16:70–2. doi: 10.12968/ijpn.2010.16.2.46752 20220684

[58] Lo SF Hayter M Hu WY Tai CY Hsu MY Li YF. Symptom burden and quality of life in patients with malignant fungating wounds. J Adv Nurs. (2012) 68:1312–21. doi: 10.1111/j.1365-2648.2011.05839.x, PMID: 22043819

[59] Lund-Nielsen B Adamsen L Kolmos HJ Rørth M Tolver A Gottrup F. The effect of honey-coated bandages compared with silver-coated bandages on treatment of malignant wounds—a randomized study. Wound Repair Regen. (2011) 19:664–70. doi: 10.1111/j.1524-475X.2011.00735.x, PMID: 22092836

[60] Grocott P. The palliative management of fungating malignant wounds. J Wound Care. (2000) 9:4–9. doi: 10.12968/jowc.2000.9.1.25942 10827661

[61] Lai YL Chang HH Huang MJ Chang KH Su WH Chen HW. Combined effect of topical arsenic trioxide and radiation therapy on skin-infiltrating lesions of breast cancer—a pilot study. Anti-Cancer Drugs. (2003) 14:825–8. doi: 10.1097/00001813-200311000-00008 14597877

[62] Lund-Nielsen B Müller K Adamsen L. Qualitative and quantitative evaluation of a new regimen for malignant wounds in women with advanced breast cancer. J Wound Care. (2005) 14:69–73. doi: 10.12968/jowc.2005.14.2.26736 15739654

[63] Tamai N Horii M Takehara K Kato S Yamamoto Y Naito A. Morphological characteristics of and factors related to moisture-associated dermatitis surrounding malignant wounds in breast cancer patients. Eur J Oncol Nurs. (2013) 17:673–80. doi: 10.1016/j.ejon.2013.05.005 23850413

[64] Schulz V Kozell K Biondo PD Stiles C Tonkin K Hagen NA. The malignant wound assessment tool: a validation study using a Delphi approach. Palliat Med. (2009) 23:266–73. doi: 10.1177/0269216309102536 19318462

[65] da Costa Santos CM de Mattos Pimenta CA Nobre MRC. A systematic review of topical treatments to control the odor of malignant fungating wounds. J Pain Symptom Manag. (2010) 39:1065–76. doi: 10.1016/j.jpainsymman.2009.11.319 20538188

[66] Chrisman CA. Care of chronic wounds in palliative care and end-of-life patients. Int Wound J. (2010) 7:214–35. doi: 10.1111/j.1742-481X.2010.00682.x 20528993 PMC7951627

[67] Clark J. Metronidazole gel in managing malodorous fungating wounds. Br J Nurs. (2002) 11:S54–60. doi: 10.12968/bjon.2002.11.Sup1.12249 11979191

[68] Dowsett C. Malignant fungating wounds: assessment and management. Br J Community Nurs. (2002) 7:394–400. doi: 10.12968/bjcn.2002.7.8.10641 12192342

[69] Naylor W. Assessment and management of pain in fungating wounds. Br J Nurs. (2001) 10:S33–6. doi: 10.12968/bjon.2001.10.Sup5.12325, PMID: 11842470

[70] Seaman S. Management of malignant fungating wounds in advanced cancer. Semin Oncol Nurs. (2006) 22:185–93. doi: 10.1016/j.soncn.2006.04.006 16893748

[71] Young CV. The effects of malodorous fungating malignant wounds on body image and quality of life. J Wound Care. (2005) 14:359–62. doi: 10.12968/jowc.2005.14.8.26827 16178290

[72] Voepel-Lewis T Zanotti J Dammeyer JA Merkel S. Reliability and validity of the face, legs, activity, cry, Consolability behavioral tool in assessing acute pain in critically ill patients. Am J Crit Care. (2010) 19:55–61. doi: 10.4037/ajcc2010624, PMID: 20045849

[73] Jensen MP. The validity and reliability of pain measures in adults with cancer. J Pain. (2003) 4:2–21. doi: 10.1054/jpai.2003.1, PMID: 14622723

[74] Ngamkham S Vincent C Finnegan L Holden JE Wang ZJ Wilkie DJ. The mcgill pain questionnaire as a multidimensional measure in people with cancer: an integrative review. Pain Manag Nurs. (2012) 13:27–51. doi: 10.1016/j.pmn.2010.12.003 22341138 PMC3285427

[75] Gauthier LR Young A Dworkin RH Rodin G Zimmermann C Warr D. Validation of the short-form McGill pain questionnaire-2 in younger and older people with cancer pain. J Pain. (2014) 15:756–70. doi: 10.1016/j.jpain.2014.04.004 24780200

[76] Warden V Hurley AC Volicer L. Development and psychometric evaluation of the pain assessment in advanced dementia (PAINAD) scale. J Am Med Dir Assoc. (2003) 4:9–15. doi: 10.1097/01.JAM.0000043422.31640.F7, PMID: 12807591

[77] Chang VT Hwang SS Feuerman M. Validation of the Edmonton symptom assessment scale. Cancer. (2000) 88:2164–71. doi: 10.1002/(sici)1097-0142(20000501)88:9<2164::aid-cncr24>3.0.co;2-5 10813730

[78] Frasier K Li V Christoforides S Daly K Loperfito A Stech K. The impact of psychosocial influences on chronic wound healing. Open J Med Psychol. (2024) 13:39–57. doi: 10.4236/ojmp.2024.133004

[79] Wilson CM McGuire DB Rodgers BL Elswick RK Temkin SM. Body image, sexuality, and sexual functioning in women with gynecologic cancer: an integrative review of the literature and implications for research. Cancer Nurs. (2021) 44:E252–86. doi: 10.1097/NCC.0000000000000818 32332264 PMC7575618

[80] Vardhan M Flaminio Z Sapru S Tilley CP Fu MR Comfort C. The microbiome, malignant Fungating wounds, and palliative care. Front Cell Infect Microbiol. (2019) 9:373. doi: 10.3389/fcimb.2019.00373, PMID: 31737576 PMC6838011

[81] Melhem SJ Nabhani-Gebara S Kayyali R. Latency of breast cancer stigma during survivorship and its influencing factors: a qualitative study. Front Oncol. (2023) 13:1075298. doi: 10.3389/fonc.2023.1075298, PMID: 36998442 PMC10043425

[82] Nagle SM Stevens KA Wilbraham SC. Wound Assessment. 2023 Jun 26. In: StatPearls [Internet]. Treasure Island (FL): Stat Pearls Publishing (2025). 29489199

[83] Lovén M Huilaja L Paananen M Torkki P. The integration of dermatology experts into primary care to assess and treat patients with skin lesions is cost-effective: a quasi-experimental study. Acad Dermatol Venereol. (2024):20451. doi: 10.1111/jdv.20451 PMC12376251 39620255

[84] The new Italian nursing code of ethics issued by the National Council of Nursing Professions (FNOPI). (2025).

[85] Capelas ML Simões ASAWF Teves CFTM Durão SAP Coelho SPF Da Silva SCFS. Indicadores de Qualidade Prioritários para os Serviços de Cuidados Paliativos em Portugal. Cad Saude Publica. (2018) 10:11–24. doi: 10.34632/CADERNOSDESAUDE.2018.7245

[86] EONS (2015). Recommendations for the care of patients with malignant fungating wounds. European Oncology Nursing Society (EONS).

[87] Dryden SV Shoemaker WG Kim JH. Wound management and nutrition for optimal wound healing. Atlas Oral Maxillofac Surg Clin. (2013) 21:37–47. doi: 10.1016/j.cxom.2012.12.008, PMID: 23498330

[88] NHS Forth Valley. Guidelines for the management of malignant wounds M:EMPHIS pathway. Heather Macgowan. (2025)

[89] Peñaranda LM. The utopia of transdisciplinary palliative care. AG Salud. (2024) 2:89. doi: 10.62486/agsalud202489

[90] Universidad Cooperativa de Colombia Bucaramanga Peñaranda Ospina LM Iglesias Meza FS Alvarado Garcia AM. ¿Podemos ver el mundo igual? interdisciplinariedad en el cuidado paliativo. Rev Cuid. (2022) 13. doi: 10.15649/cuidarte.2568 PMC11290781 40114800

[91] Pramod S Dumville J Norman G Stringer J. A survey of UK nurses about their care of people with malignant fungating wounds. Eur J Oncol Nurs. (2024) 70:102609. doi: 10.1016/j.ejon.2024.102609, PMID: 38810584

[92] Streiner D. Health measurement scales: a practical guide to their development and use (5th edition). Aust N Z J Public Health. (2016) 40:294–5. doi: 10.1111/1753-6405.12492 27242256

[93] Park H Kim KE Moon E Kang T. Psychometric properties of assessment tools for depression, anxiety, distress, and psychological problems in breast Cancer patients: a systematic review. Psychiatry Investig. (2023) 20:395–407. doi: 10.30773/pi.2022.0316, PMID: 37253465 PMC10232053

[94] Fauci AJ Coclite D Napoletano A D'Angelo D Biffi A Castellini G. Clinical practice guideline for the integrated management of major trauma by the Italian National Institute of health: process and methods. Ann Ist Super Sanita. (2021) 57:343–51. doi: 10.4415/ANN_21_04_09, PMID: 35076424

